# Supplementary material for: Stability of Ophthalmic Atropine Solutions for Child Myopia Control
Source: Pharmaceutics. 2020 Aug 17;12(8):781. doi: 10.3390/pharmaceutics12080781 (PMC7465901; doi:10.3390/pharmaceutics12080781)
Supplement: Supplementary file 1 [file pharmaceutics-12-00781-s001.pdf]

pharmaceutics

Supplementary Materials: Stability of Ophthalmic Atropine Solutions for Child Myopia Control

Baptiste Berton, Philip Chennell, Mouloud Yessaad, Yassine Bouattour, Mireille Jouannet, Mathieu Wasiak and Valérie Sautou

Validation of Tropic Acid Quantification Method

The chromatographic method used was found linear for concentrations ranging from 0.1 to 5 µg/mL. Average regression equation was $y = 41722x - 439$ where x is the tropic acid concentration (in µg/mL) and y the surface area of the corresponding chromatogram peak. Interception was not significantly different from zero and average determination coefficient R^2 of three calibration curves was 0.9998.

The relative mean trueness bias coefficients were of less than 2%. Mean repeatability RSD coefficients was of 1.32%, and mean intermediate precision RSD coefficients was of 3.26%. The accuracy profile constructed with the data showed that the limits of 95% confidence interval coefficients were all within 6% of the expected value, except for the 0.10 µg/mL calibration point for which the lower range limit was of −6.4%.

Complementary Study: Quantification of Tropic Acid in Commercial Ophthalmic Atropine Solutions

As a complementary study, the principal BP of atropine (tropic acid) was quantified in two commercial atropine ophthalmic solutions: 0.5% atropine (5 mg/mL) in 10 mL multidose eyedropper (Alcon) and 1% atropine (10 mg/mL) in 0.4 mL single dose eyedropper (Faure). 1/50th and 1/100th dilutions of the two commercial solutions were realized to reach the experimental atropine concentration of 0.1 mg/mL. Tropic acid presence and quantification were evaluated with the same method describe further for atropine quantification and BPs research. The tests were performed in triplicate.

Two commercial atropine ophthalmic formulations presented high concentrations of tropic acid, as presented in Table 1. The concentrations of tropic acid were 6 to 11 times higher than those found in the studied atropine formulations after 6 months conservation (21.4 and 40.2 µg/mL *versus* 3.5 µg/mL).

Table 1. Atropine and tropic acid concentrations in commercial atropine ophthalmic formulations.

	**Atropine concentration (mg/mL)**			**Tropic acid concentration (µg/ml)**		
0.5% atropine in 10 mL multidose eyedropper (Alcon)	5.3	±	0.0	21.4	±	0.4
1% atropine in 0.4 mL single dose eyedropper (Faure)	10.4	±	0.1	40.2	±	0.6

Raw data

Raw data is presented in the following tables (Tables 2 to 6).

Table 2. Atropine and tropic acid quantification.

		**Atropine AUC**		**Atropine Concentration (mg/mL)**		**Tropic acid AUC**		**Tropic acid Concentration (µg/mL)**	
	**Nb of unit**	**Gamma PEBD**	**EO PEBD**	**Gamma PEBD**	**EO PEBD**	**Gamma PEBD**	**EO PEBD**	**Gamma PEBD**	**EO PEBD**
Day 0	1	2245774	2251614	0.10033	0.10059	NA	NA	NA	NA
	2	2252215	2252040	0.10062	0.10061	NA	NA	NA	NA
	3	2244406	2252105	0.10026	0.10061	NA	NA	NA	NA
	4	2246283	2252202	0.10035	0.10061	NA	NA	NA	NA
	5	2250389	2250062	0.10053	0.10052	NA	NA	NA	NA
Day 8	1	2237327	2246380	0.09995	0.10035	5952	5968	0.2	0.2
	2	2238204	2245525	0.09999	0.10031	5708	5863	0.1	0.2
	3	2242888	2245311	0.10020	0.10031	5965	6041	0.2	0.2
	4	2239986	2248017	0.10007	0.10043	5966	5847	0.2	0.2
	5	2239946	2247185	0.10006	0.10039	5981	5756	0.2	0.1
Day 15	1	2231738	2237453	0.09969	0.09995	11990	11421	0.3	0.3
	2	2230922	2238453	0.09966	0.10000	11590	11533	0.3	0.3
	3	2232445	2239359	0.09973	0.10004	12041	11411	0.3	0.3
	4	2230975	2238497	0.09966	0.10000	11839	11526	0.3	0.3
	5	2232593	2238647	0.09973	0.10001	12366	11509	0.3	0.3
Day 30	1	2228302	2230630	0.09954	0.09964	26059	24139	0.6	0.6
	2	2225530	2232078	0.09942	0.09971	25374	24596	0.6	0.6
	3	2223462	2231739	0.09932	0.09969	24246	23747	0.6	0.6
	4	2224868	2232857	0.09939	0.09975	25742	24431	0.6	0.6
	5	2230460	2232593	0.09964	0.09973	24833	24947	0.6	0.6
Day 60	1	2200643	2210560	0.09830	0.09874	52400	49334	1.3	1.2
	2	2205060	2210609	0.09850	0.09874	52172	50913	1.3	1.2
	3	2205771	2211835	0.09853	0.09880	50528	51098	1.2	1.2
	4	2203923	2213348	0.09844	0.09887	51251	50562	1.2	1.2
	5	2200295	2212743	0.09828	0.09884	51952	50715	1.3	1.2
Day 90	1	2167052	2181056	0.09679	0.09742	92245	86953	2.2	2.1
	2	2163396	2174140	0.09662	0.09710	92800	90032	2.2	2.2
	3	2163286	2183164	0.09662	0.09751	93957	87432	2.3	2.1
	4	2164854	2177172	0.09669	0.09724	92657	90808	2.2	2.2
	5	2165957	2177253	0.09674	0.09724	92933	88251	2.2	2.1
Day 180	1	2116721	2133153	0.09452	0.09526	144155	143703	3.5	3.5
	2	2123068	2137217	0.09481	0.09544	144880	142454	3.5	3.4
	3	2122013	2135924	0.09476	0.09539	143250	144048	3.4	3.5
	4	2122672	2137014	0.09479	0.09544	151728	140567	3.6	3.4
	5	2117772	2135585	0.09457	0.09537	142547	142030	3.4	3.4

Table 3. Visual aspect and chromaticity data.

Day							
Day 0		**Gamma LDPE Unit 1**	**Gamma LDPE Unit 2**	**Gamma LDPE Unit 3**	**Gamma LDPE Unit 4**	**Gamma LDPE Unit 5**	**Mean**
	Visual aspect	Visual examination: colourless, clear, without particles					
	Luminance (Y)	100.33	100.35	99.91	100.35	99.92	100.172
	Chromaticity (x)	0.3127	0.3127	0.3128	0.3127	0.3127	0.31272
	Chromaticity (y)	0.3291	0.3291	0.3291	0.3291	0.329	0.32908
	Excitation percentage Pe (%)	0.02	0	0.03	0.01	0.01	0.014
		**EO LDPE Unit 1**	**EO LDPE Unit 2**	**EO LDPE Unit 3**	**EO LDPE Unit 4**	**EO LDPE Unit 5**	**Mean**
	Visual aspect	Visual examination: colourless, clear, without particles					
	Luminance (Y)	99.91	100.3	100.25	100.36	100.17	100.198
	Chromaticity (x)	0.3127	0.3127	0.3127	0.3127	0.3128	0.31272
	Chromaticity (y)	0.329	0.329	0.3291	0.3291	0.3291	0.32906
	Excitation percentage Pe (%)	0.01	0	0.02	0.01	0.03	0.014
Day 8	*Solutions*	**Gamma LDPE Unit 1**	**Gamma LDPE Unit 2**	**Gamma LDPE Unit 3**	**Gamma LDPE Unit 4**	**Gamma LDPE Unit 5**	**Mean**
	Visual aspect	Visual examination: colourless, clear, without particles					
	Luminance (Y)	99.96	100.02	99.98	100.02	99.91	99.978
	Chromaticity (x)	0.3127	0.3127	0.3126	0.3126	0.3127	0.31266
	Chromaticity (y)	0.329	0.329	0.329	0.3291	0.329	0.32902
	Excitation percentage Pe (%)	0.03	0.01	0.04	0.03	0.01	0.024
		**EO LDPE Unit 1**	**EO LDPE Unit 2**	**EO LDPE Unit 3**	**EO LDPE Unit 4**	**EO LDPE Unit 5**	**Mean**
	Visual aspect	Visual examination: colourless, clear, without particles					
	Luminance (Y)	99.98	99.98	99.93	100	99.94	99.966
	Chromaticity (x)	0.3127	0.3127	0.3127	0.3127	0.3127	0.3127
	Chromaticity (y)	0.329	0.329	0.329	0.329	0.329	0.329
	Excitation percentage Pe (%)	0.01	0	0.01	0.01	0.01	0.008
Day 15		**Gamma LDPE Unit 1**	**Gamma LDPE Unit 2**	**Gamma LDPE Unit 3**	**Gamma LDPE Unit 4**	**Gamma LDPE Unit 5**	**Mean**
	Visual aspect	Visual examination: colourless, clear, without particles					
	Luminance (Y)	99.72	99.96	99.97	99.91	99.73	99.858
	Chromaticity (x)	0.3127	0.3126	0.3126	0.3126	0.3126	0.31262
	Chromaticity (y)	0.329	0.3289	0.3289	0.329	0.3289	0.32894
	Excitation percentage Pe (%)	0.02	0.04	0.05	0.04	0.04	0.038
		**EO LDPE Unit 1**	**EO LDPE Unit 2**	**EO LDPE Unit 3**	**EO LDPE Unit 4**	**EO LDPE Unit 5**	**Mean**
	Visual aspect	Visual examination: colourless, clear, without particles					

Day	Parameter	Unit 1	Unit 2	Unit 3	Unit 4	Unit 5	Mean
	Luminance (Y)	99.81	99.88	99.79	99.72	99.83	99.806
	Chromaticity (x)	0.3127	0.3126	0.3127	0.3126	0.3126	0.31264
	Chromaticity (y)	0.329	0.329	0.329	0.3289	0.3289	0.32896
	Excitation percentage Pe (%)	0.02	0.03	0.02	0.07	0.05	0.038
**Day 30**		**Gamma LDPE Unit 1**	**Gamma LDPE Unit 2**	**Gamma LDPE Unit 3**	**Gamma LDPE Unit 4**	**Gamma LDPE Unit 5**	**Mean**
	Visual aspect	Visual examination: colourless, clear, without particles					
	Luminance (Y)	100.4	100.48	100.19	100.09	100.29	100.29
	Chromaticity (x)	0.3126	0.3125	0.3125	0.3125	0.3125	0.31252
	Chromaticity (y)	0.3289	0.3289	0.3288	0.3288	0.3288	0.32884
	Excitation percentage Pe (%)	0.05	0.08	0.09	0.1	0.11	0.086
		**EO LDPE Unit 1**	**EO LDPE Unit 2**	**EO LDPE Unit 3**	**EO LDPE Unit 4**	**EO LDPE Unit 5**	**Mean**
	Visual aspect	Visual examination: colourless, clear, without particles					
	Luminance (Y)	100.07	100.35	100.32	100.17	100.36	100.254
	Chromaticity (x)	0.3126	0.3126	0.3125	0.3125	0.3125	0.31254
	Chromaticity (y)	0.3287	0.3289	0.3289	0.3289	0.3289	0.32886
	Excitation percentage Pe (%)	0.09	0.07	0.08	0.09	0.11	0.088
**Day 60**		**Gamma LDPE Unit 1**	**Gamma LDPE Unit 2**	**Gamma LDPE Unit 3**	**Gamma LDPE Unit 4**	**Gamma LDPE Unit 5**	**Mean**
	Visual aspect	Visual examination: colourless, clear, without particles					
	Luminance (Y)	99.86	99.85	99.87	99.66	99.77	99.802
	Chromaticity (x)	0.3127	0.3128	0.3127	0.3127	0.3127	0.31272
	Chromaticity (y)	0.329	0.3292	0.329	0.329	0.329	0.32904
	Excitation percentage Pe (%)	0.01	0.05	0.01	0	0.01	0.016
		**EO LDPE Unit 1**	**EO LDPE Unit 2**	**EO LDPE Unit 3**	**EO LDPE Unit 4**	**EO LDPE Unit 5**	**Mean**
	Visual aspect	Visual examination: colourless, clear, without particles					
	Luminance (Y)	99.83	99.86	99.88	99.77	99.8	99.828
	Chromaticity (x)	0.3127	0.3127	0.3127	0.3127	0.3129	0.31274
	Chromaticity (y)	0.329	0.329	0.3291	0.329	0.3291	0.32904
	Excitation percentage Pe (%)	0.01	0.01	0.02	0.01	0.05	0.02
**Day 90**		**Gamma LDPE Unit 1**	**Gamma LDPE Unit 2**	**Gamma LDPE Unit 3**	**Gamma LDPE Unit 4**	**Gamma LDPE Unit 5**	**Mean**
	Visual aspect	Visual examination: colourless, clear, without particles					
	Luminance (Y)	99.82	99.85	99.89	99.87	99.84	99.854
	Chromaticity (x)	0.3128	0.3128	0.3128	0.3128	0.3128	0.3128

	Chromaticity (y)	0.3291	0.3291	0.3291	0.3291	0.3291	0.329 1
	Excitation percentage Pe (%)	0.05	0.06	0.06	0.05	0.05	0.054
	Solutions	**EO LDPE Unit 1**	**EO LDPE Unit 2**	**EO LDPE Unit 3**	**EO LDPE Unit 4**	**EO LDPE Unit 5**	**Mea n**
	Visual aspect	Visual examination: colourless, clear, without particles					
	Luminance (Y)	99.2	99.39	99.42	99.4	99.81	99.44 4
	Chromaticity (x)	0.3126	0.3128	0.3127	0.3128	0.3127	0.312 72
	Chromaticity (y)	0.3291	0.3292	0.3291	0.3291	0.3291	0.329 12
	Excitation percentage Pe (%)	0.03	0.09	0.02	0.05	0.02	0.042
**Day 180**		**Gamma LDPE Unit 1**	**Gamma LDPE Unit 2**	**Gamma LDPE Unit 3**	**Gamma LDPE Unit 4**	**Gamma LDPE Unit 5**	**Mea n**
	Visual aspect	Visual examination: colourless, clear, without particles					
	Luminance (Y)	99.18	99.28	99.67	100.01	100.45	99.71 8
	Chromaticity (x)	0.313	0.313	0.3127	0.3127	0.3126	0.312 8
	Chromaticity (y)	0.3293	0.3294	0.3291	0.3291	0.329	0.329 18
	Excitation percentage Pe (%)	0.17	0.17	0.02	0.03	0.04	0.086
		**EO LDPE Unit 1**	**EO LDPE Unit 2**	**EO LDPE Unit 3**	**EO LDPE Unit 4**	**EO LDPE Unit 5**	**Mea n**
	Visual aspect	Visual examination: colourless, clear, without particles					
	Luminance (Y)	100.46	100.34	100.39	100.47	100.48	100.4 28
	Chromaticity (x)	0.3125	0.3126	0.3126	0.3126	0.3126	0.312 58
	Chromaticity (y)	0.3289	0.3289	0.329	0.3289	0.329	0.328 94
	Excitation percentage Pe (%)	0.08	0.06	0.05	0.06	0.05	0.06

Table 4. Complete pH data of atropine solutions.

Day 0		Gamma LDPE Unit 1	Gamma LDPE Unit 2	Gamma LDPE Unit 3	Gamma LDPE Unit 4	Gamma LDPE Unit 5	Mean
	pH	6.09	6.1	6.1	6.1	6.09	6.096
		EO LDPE Unit 1	EO LDPE Unit 2	EO LDPE Unit 3	EO LDPE Unit 4	EO LDPE Unit 5	Mean
	pH	6.08	6.1	6.09	6.1	6.1	6.094
Day 8		Gamma LDPE Unit 1	Gamma LDPE Unit 2	Gamma LDPE Unit 3	Gamma LDPE Unit 4	Gamma LDPE Unit 5	Mean
	pH	6.1	6.09	6.11	6.11	6.12	6.106
		EO LDPE Unit 1	EO LDPE Unit 2	EO LDPE Unit 3	EO LDPE Unit 4	EO LDPE Unit 5	Mean
	pH	6.1	6.1	6.11	6.11	6.11	6.106
Day 15		Gamma LDPE Unit 1	Gamma LDPE Unit 2	Gamma LDPE Unit 3	Gamma LDPE Unit 4	Gamma LDPE Unit 5	Mean
	pH	6.11	6.12	6.12	6.16	6.14	6.13
		EO LDPE Unit 1	EO LDPE Unit 2	EO LDPE Unit 3	EO LDPE Unit 4	EO LDPE Unit 5	Mean
	pH	6.14	6.13	6.13	6.12	6.13	6.13
Day 30		Gamma LDPE Unit 1	Gamma LDPE Unit 2	Gamma LDPE Unit 3	Gamma LDPE Unit 4	Gamma LDPE Unit 5	Mean
	pH	6.11	6.12	6.13	6.13	6.14	6.126
		EO LDPE Unit 1	EO LDPE Unit 2	EO LDPE Unit 3	EO LDPE Unit 4	EO LDPE Unit 5	Mean
	pH	6.13	6.12	6.15	6.16	6.15	6.142
Day 60		Gamma LDPE Unit 1	Gamma LDPE Unit 2	Gamma LDPE Unit 3	Gamma LDPE Unit 4	Gamma LDPE Unit 5	Mean
	pH	6.11	6.14	6.13	6.13	6.14	6.13
		EO LDPE Unit 1	EO LDPE Unit 2	EO LDPE Unit 3	EO LDPE Unit 4	EO LDPE Unit 5	Mean
	pH	6.12	6.13	6.13	6.13	6.13	6.128
Day 90		Gamma LDPE Unit 1	Gamma LDPE Unit 2	Gamma LDPE Unit 3	Gamma LDPE Unit 4	Gamma LDPE Unit 5	Mean
	pH	6.16	6.2	6.22	6.23	6.23	6.208
		EO LDPE Unit 1	EO LDPE Unit 2	EO LDPE Unit 3	EO LDPE Unit 4	EO LDPE Unit 5	Mean
	pH	6.21	6.21	6.2	6.21	6.2	6.206
Day 180		Gamma LDPE Unit 1	Gamma LDPE Unit 2	Gamma LDPE Unit 3	Gamma LDPE Unit 4	Gamma LDPE Unit 5	Mean
	pH	6.12	6.12	6.12	6.13	6.13	6.124
		EO LDPE Unit 1	EO LDPE Unit 2	EO LDPE Unit 3	EO LDPE Unit 4	EO LDPE Unit 5	Mean
	pH	6.1	6.1	6.08	6.09	6.08	6.09

Table 5. Complete osmolality (mOsmol/kg) of atropine solutions.

Day 0	Gamma LDPE Unit 1	Gamma LDPE Unit 2	Gamma LDPE Unit 3	Gamma LDPE Unit 4	Gamma LDPE Unit 5	Mean
	399	428	415	398	420	412
	EO LDPE Unit 1	EO LDPE Unit 2	EO LDPE Unit 3	EO LDPE Unit 4	EO LDPE Unit 5	Mean
	401	399	399	397	398	398.8
Day 8	Gamma LDPE Unit 1	Gamma LDPE Unit 2	Gamma LDPE Unit 3	Gamma LDPE Unit 4	Gamma LDPE Unit 5	Mean
	407	397	397	397	403	400.2
	EO LDPE Unit 1	EO LDPE Unit 2	EO LDPE Unit 3	EO LDPE Unit 4	EO LDPE Unit 5	Mean
	398	396	409	402	399	400.8
Day 15	Gamma LDPE Unit 1	Gamma LDPE Unit 2	Gamma LDPE Unit 3	Gamma LDPE Unit 4	Gamma LDPE Unit 5	Mean
	390	396	412	416	400	402.8
	EO LDPE Unit 1	EO LDPE Unit 2	EO LDPE Unit 3	EO LDPE Unit 4	EO LDPE Unit 5	Mean
	417	404	407	410	406	408.8
Day 30	Gamma LDPE Unit 1	Gamma LDPE Unit 2	Gamma LDPE Unit 3	Gamma LDPE Unit 4	Gamma LDPE Unit 5	Mean
	378	402	385	401	400	393.2
	EO LDPE Unit 1	EO LDPE Unit 2	EO LDPE Unit 3	EO LDPE Unit 4	EO LDPE Unit 5	Mean
	402	405	405	405	406	404.6
Day 60	Gamma LDPE Unit 1	Gamma LDPE Unit 2	Gamma LDPE Unit 3	Gamma LDPE Unit 4	Gamma LDPE Unit 5	Mean
	399	397	407	399	398	400
	EO LDPE Unit 1	EO LDPE Unit 2	EO LDPE Unit 3	EO LDPE Unit 4	EO LDPE Unit 5	Mean
	420	428	422	401	402	414.6
Day 90	Gamma LDPE Unit 1	Gamma LDPE Unit 2	Gamma LDPE Unit 3	Gamma LDPE Unit 4	Gamma LDPE Unit 5	Mean
	409	408	416	428	405	413.2
	EO LDPE Unit 1	EO LDPE Unit 2	EO LDPE Unit 3	EO LDPE Unit 4	EO LDPE Unit 5	Mean
	409	403	406	421	401	408
Day 180	Gamma LDPE Unit 1	Gamma LDPE Unit 2	Gamma LDPE Unit 3	Gamma LDPE Unit 4	Gamma LDPE Unit 5	Mean
	401	433	399	441	417	418.2
	EO LDPE Unit 1	EO LDPE Unit 2	EO LDPE Unit 3	EO LDPE Unit 4	EO LDPE Unit 5	Mean
	402	415	405	405	399	405.2

Table 6. Complete turbidy (FNU, formazine nephelometric unit) of atropine solutions performed on pooled volume of 5 units.

Day 0	Gamma LDPE Unit 1	Gamma LDPE Unit 2	Gamma LDPE Unit 3	Gamma LDPE Unit 4	Gamma LDPE Unit 5
	0.33				
	EO LDPE Unit 1	EO LDPE Unit 2	EO LDPE Unit 3	EO LDPE Unit 4	EO LDPE Unit 5
	0.32				
Day 8	Gamma LDPE Unit 1	Gamma LDPE Unit 2	Gamma LDPE Unit 3	Gamma LDPE Unit 4	Gamma LDPE Unit 5
	0.31				
	EO LDPE Unit 1	EO LDPE Unit 2	EO LDPE Unit 3	EO LDPE Unit 4	EO LDPE Unit 5
	0.31				
Day 15	Gamma LDPE Unit 1	Gamma LDPE Unit 2	Gamma LDPE Unit 3	Gamma LDPE Unit 4	Gamma LDPE Unit 5
	0.27				
	EO LDPE Unit 1	EO LDPE Unit 2	EO LDPE Unit 3	EO LDPE Unit 4	EO LDPE Unit 5
	0.26				
Day 30	Gamma LDPE Unit 1	Gamma LDPE Unit 2	Gamma LDPE Unit 3	Gamma LDPE Unit 4	Gamma LDPE Unit 5
	0.78				
	EO LDPE Unit 1	EO LDPE Unit 2	EO LDPE Unit 3	EO LDPE Unit 4	EO LDPE Unit 5
	0.54				
Day 60	Gamma LDPE Unit 1	Gamma LDPE Unit 2	Gamma LDPE Unit 3	Gamma LDPE Unit 4	Gamma LDPE Unit 5
	0.78				
	EO LDPE Unit 1	EO LDPE Unit 2	EO LDPE Unit 3	EO LDPE Unit 4	EO LDPE Unit 5
	0.44				
Day 90	Gamma LDPE Unit 1	Gamma LDPE Unit 2	Gamma LDPE Unit 3	Gamma LDPE Unit 4	Gamma LDPE Unit 5
	0.43				
	EO LDPE Unit 1	EO LDPE Unit 2	EO LDPE Unit 3	EO LDPE Unit 4	EO LDPE Unit 5
	0.34				
Day 180	Gamma LDPE Unit 1	Gamma LDPE Unit 2	Gamma LDPE Unit 3	Gamma LDPE Unit 4	Gamma LDPE Unit 5
	0.93				
	EO LDPE Unit 1	EO LDPE Unit 2	EO LDPE Unit 3	EO LDPE Unit 4	EO LDPE Unit 5
	0.64				
